# Proteomic Analysis of the Function of a Novel Cold-Regulated Multispanning Transmembrane Protein COR413-PM1 in *Arabidopsis*

**DOI:** 10.3390/ijms19092572

**Published:** 2018-08-29

**Authors:** Chen Su, Kai Chen, Qingqian Ding, Yongying Mou, Rui Yang, Mengjie Zhao, Bo Ma, Zhaoshi Xu, Youzhi Ma, Yinghong Pan, Ming Chen, Yajun Xi

**Affiliations:** 1 College of Agronomy, Northwest A&F University, Yangling 712100, China; suchen1213@163.com (C.S.); 18334534481@163.com (R.Y.); 2 Institute of Crop Sciences, Chinese Academy of Agricultural Sciences (CAAS)/National Key Facility for Crop Gene Resources and Genetic Improvement, Beijing 100081, China; chenkai8@vip.163.com (K.C.); dingqingqian0215@163.com (Q.D.); mu_yongying@163.com (Y.M.); zhao_mengjie0815@163.com (M.Z.); mabo8686@163.com (B.M.); xuzhaoshi@caas.cn (Z.X.); mayouzhi@caas.cn (Y.M.); 3 Key Laboratory of Biology and Genetic Improvement of *Triticeae* Crops, Ministry of Agriculture, Beijing 100081, China; 4 College of Life Science, Jilin Agricultural University, Changchun 130118, China

**Keywords:** freezing stress, *COR413-PM1*, gene function, proteomic analysis, *Arabidopsis*

## Abstract

The plasma membrane is the first subcellular organ that senses low temperature, and it includes some spanning transmembrane proteins that play important roles in cold regulation. COR413-PM1 is a novel multispanning transmembrane cold-regulated protein; however, the related functions are not clear in *Arabidopsis*. We found the tolerance to freezing stress of *cor413-pm1* was lower than wild-type (WT). A proteomics method was used to analyze the differentially abundant proteins (DAPs) between *cor413-pm1* and WT. A total of 4143 protein groups were identified and 3139 were accurately quantitated. The DAPs associated with *COR413-PM1* and freezing treatment were mainly involved in the metabolism of fatty acids, sugars, and purine. Quantitative real-time PCR (qRT-PCR) confirmed the proteomic analysis results of four proteins: fatty acid biosynthesis 1 (FAB1) is involved in fatty acid metabolism and might affect the plasma membrane structure; fructokinase 3 (FRK3) and sucrose phosphate synthase A1 (SPSA1) play roles in sugar metabolism and may influence the ability of osmotic adjustment under freezing stress; and GLN phosphoribosyl pyrophosphate amidotransferase 2 (ASE2) affects freezing tolerance through purine metabolism pathways. In short, our results demonstrate that the multispanning transmembrane protein COR413-PM1 regulates plant tolerance to freezing stress by affecting the metabolism of fatty acids, sugars, and purine in *Arabidopsis*.

## 1. Introduction

Cold stress tolerance is essential for plant development and environmental acclimation. Cold stress refers to chilling (0–20 °C) and/or freezing (<0 °C) temperatures that can induce ice formation in plant tissues, damage the normal function and structure of cells, and limit plant growth and development, resulting in significant losses in agricultural production [1,2]. Plants acquiring cold stress tolerance upon prior exposure to low nonfreezing temperatures is known as cold acclimation. Cold acclimation involves numerous changes in structures specific to the plasma membrane, the reprogramming of regulated metabolism, and gene expression [3,4,5]. Significant progress has been made in the past several decades in describing the plant transcriptome regulating network associated with cold stress from exposure to chilling and freezing temperatures, and this work has significantly contributed to our understanding of tolerance mechanisms to cold stress [1]. The *C-repeat Binding Factor/DRE Binding Factor* (*CBF/DREB*) controls the expression of cold-induced genes, and its transcriptional regulatory cascade is widely recognized as the main cold signaling pathway [4]. The *CBF* transcription factors (*CBF1/DREB1b*, *CBF2/DREB1c*, and *CBF3/DREB1a*) together with an *APETALA2/ethylene response factor* (*AP2*) DNA binding domain play a key role in the cold signaling pathway in plants [6,7]. This signal transduction causes a series of downstream responses to cold. After exposure to cold stress, except for transcriptional level changes, plants make large changes in their physiological and biochemical metabolisms [1], such as metabolism of carbohydrates, proteins, and amino acids, and in signal transduction [8,9]. In *Arabidopsis*, the downregulation of photosynthesis and hormonal responses is associated with enhanced freezing tolerance [10]. The accumulation of soluble sugars is a fundamental component of enhanced freezing tolerance [11]. On the other hand, the signaling system of low temperature acclimation crosses with other signal systems, such as those of drought or high salt stress, due to the fact that they share many common features including cellular dehydration, which can cause injury to plants after exposure to freezing temperatures, drought, or salt [11,12]. Plants have evolved various mechanisms for cold sensing and stress signal transduction, and these varied mechanisms interact to produce cold tolerance.

In the process of cold acclimation in plants, the expression of cold response genes plays an important role [13]. A study using an Affymetrix GeneChips assay showed that 3.9% of genes in *Arabidopsis* were determined to be cold responsive, with 655 upregulated and 284 downregulated under low-temperature conditions [14]. The expression of cold-regulated (COR) genes can be induced by low temperatures [15], while some genes are also induced by drought and abscisic acid (ABA) [16]. The expression of COR gene *COR29A*, which is present in *Arabidopsis*, is induced not only by cold but also by drought stress and ABA treatment [17]. *BnCOR25* is significantly induced by both cold stress and ABA treatment in *Brassica napus* [18]. At present, most of the genes involved in plant response to low-temperature stress are located in the cytoplasm or nucleus, and a few studies have reported membrane localization proteins involved in plants’ response to low temperatures. The COR413-like protein containing five putative transmembrane domains (TMD) belongs to a subfamily of the COR protein family. The *COR413* gene subfamily encodes two distinct groups of multispanning transmembrane proteins including COR413-PM proteins targeted to the plasma membrane and COR413-TM proteins targeted to the thylakoid membrane [19]. *COR413* is not only induced by freezing temperatures but also by water stress and ABA treatment [20]. Low-temperature stress can influence the composition of the plasma membrane by reducing cellular membrane fluidity and increasing rigidity [21]. The structural changes associated with plasma membrane rigidification can lead to the expression of COR and cold-acclimation-related genes in alfalfa (*Medicago sativa*) and *B. napus* [21,22]. For example, for two late embryogenesis abundant (LEA) proteins, COR15A and COR15B, expression is highly cold induced, and their proteins are able to interact with membranes and act as membrane protectants [23]. The expression of *COR15A*, as a hydrophilic protein gene, increases freezing tolerance in the chloroplasts of nonacclimated plants [24]. In addition, *COR47* is closely associated with frost tolerance and can act as an anti-dehydrating agent to prevent excessive dehydration damage to plant cells caused by low temperatures [25]. Accumulation of the dehydrin *WCOR410* is associated with the capacity for high freezing tolerance in wheat (*Triticum aestivum* Linn.) [26]. Studies of the functions and regulated mechanisms of COR proteins, especially of the plasma membrane localization COR proteins, are helpful for understanding the relationship between the plasma membrane localization protein system and the tolerance to low-temperature stress.

To identify the gene function of *COR413-PM1* under freezing stress, we obtained the *Arabidopsis* mutant *cor413-pm1* and compared freezing stress tolerance between mutant and wild-type (WT) plants. Proteins are the major players in most cellular events, and unlike transcripts, proteins are the direct effectors of plant stress response. Thus, an investigation of changes in plant proteomes is highly important, and proteome analysis could help uncover additional novel proteins that respond to low temperatures [27]. Therefore, a proteomic analysis on seedling shoots of the mutant *cor413-pm1* and its WT was performed. In this study, 61 differentially abundant proteins (DAPs) associated with *COR413-PM1* and 180 DAPs associated with freezing treatment were identified as being involved in physiological processes including the metabolisms of fatty acids, fructose and mannose, starch and sucrose, purine, amino sugars, and nucleotide sugars.

## 2. Results

### 2.1. Gene Functions of COR413-PM1

**Figure 1:**
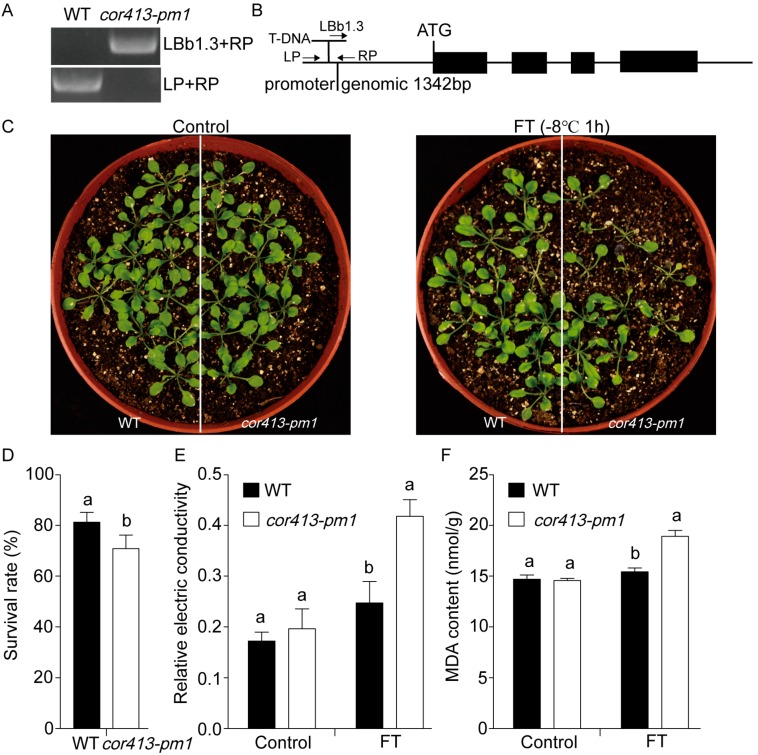
Identification of the mutant *cor413-pm1* and growth phenotypes of the *cor413-pm1* and wild-type (WT) exposed to the freezing treatment (FT). (**A**) Identification of the homozygous mutant *cor413-pm1*. (**B**) Insertion site of transfer DNA (T-DNA) in the promoter region of the mutant *cor413-pm1*. The black box represents the exons. (**C**) Freezing stress tolerance assay of *cor413-pm1* and WT. (**D**) Comparison of survival rates between *cor413-pm1* and WT after exposure to the freezing treatment. Error bars indicate standard deviation (SD) (*n* = 4) (**E**) Relative electrical conductivity and (**F**) malondialdehyde (MDA) content of *cor413-pm1* compared with those of the WT. Error bars indicate the standard error of the mean (SEM) (*n* = 3). Different letters in (**D**–**F**) indicate significant differences between WT and *cor413-pm1* at *p* < 0.05.

The expression patterns of *COR413-PM1* under 4 °C in WT are shown in Supplementary Figure S1A. The expression of *COR413-PM1* was upregulated; its maximum expression was 12.43-fold greater after 48 h of cold treatment. To identify the homozygous genotype of the transfer DNA (T-DNA) insertion mutant *cor413-pm1*, the DNA extractions of the mutant *cor413-pm1* and WT were used as templates for amplification using the insertion site-specific primers of *cor413-pm1* (T-DNA was inserted into the promoter region of *COR413-PM1* gene shown in Figure 1B) and T-DNA border primer (LBb1.3) [28,29]. Primers used for mutant identification are listed in Supplementary Table S1. The fragment containing the T-DNA right border and part of the promoter of the *COR413-PM1* gene could be amplified in the mutant *cor413-pm1*, whereas the fragment containing only the promoter of the *COR413-PM1* gene could not be amplified in the mutant *cor413-pm1* (Figure 1A). The result confirmed the homozygous genotype of the T-DNA insertion mutant *cor413-pm1*. In addition, the homozygous mutant, *cor413-pm1*, was confirmed through reverse transcription PCR (RT-PCR) and quantitative real-time PCR (qRT-PCR). Nothing was detected in the mutant *cor413-pm1* by the qCOR413-PM1 primers. The relative expression level of *COR413-PM1* in the *cor413-pm1* was only 10.8% of WT (Supplementary Figure S1B). The PCR product of the mutant *cor413-pm1* was sequenced and confirmed with the *COR413-PM1* (AT2G15970) genome sequence from the *Arabidopsis* Information Resource (TAIR) database (Available online: http://www.arabidopsis.org/). These results demonstrate that the T-DNA gene is inserted into the promoter region of *COR413-PM1* gene in *cor413-pm1* mutant, resulting in a significant decrease in *COR413-PM1* gene expression. Then, the freezing stress tolerance of transgenic *cor413-pm1* plants was investigated. The WT and *cor413-pm1* mutant seedlings were exposed to −8 °C for 1 h in a refrigerator (0.9 × 0.7 × 1.3 m^3^). The phenotype of the mutant *cor413-pm1* seedlings was similar to that of the WT at normal temperature (Figure 1C), whereas the seedlings of *cor413-pm1* were more sensitive to freezing stress than those of the WT at freezing temperature (−8 °C). Leaves of the mutant *cor413-pm1* seedlings turned yellow and then died (Figure 1C), while the negative effects were not presented in the leaves of WT seedlings. The survival rate of the *cor413-pm1* (70.83%) was significantly lower than that of the WT (81.25%) (*p* < 0.05) (Figure 1D). Two physiological properties were investigated to evaluate tolerance to freezing stress: relative electrical conductivity and malondialdehyde (MDA) content. Relative electrical conductivity was used as an indicator of plasma membrane damage caused by freezing temperatures [30]. The results showed that relative electrical conductivity of all plants increased after exposure to the freezing treatment; relative electrical conductivity in *cor413-pm1* was significantly higher than that in the WT (*p* < 0.05) (Figure 1E). Amounts of reactive oxygen species caused by the lipid peroxidation process increases under stress conditions, and MDA content is a typical indicator of membrane lipid peroxidation [31]. The MDA content of *cor413-pm1* was greater by 22.66% of that of the WT and reached 18.92 nmol/g after exposure to the freezing treatment (*p* < 0.05) (Figure 1F). These results showed that the membrane damage of *cor413-pm1* mutant was more severe than WT at low temperatures and tolerance to low-temperature stress was significantly lower than that of WT.

### 2.2. Identification of Differentially Abundant Proteins (DAPs) in Seedling Shoots of cor413-pm1 and WT

**Figure 2:**
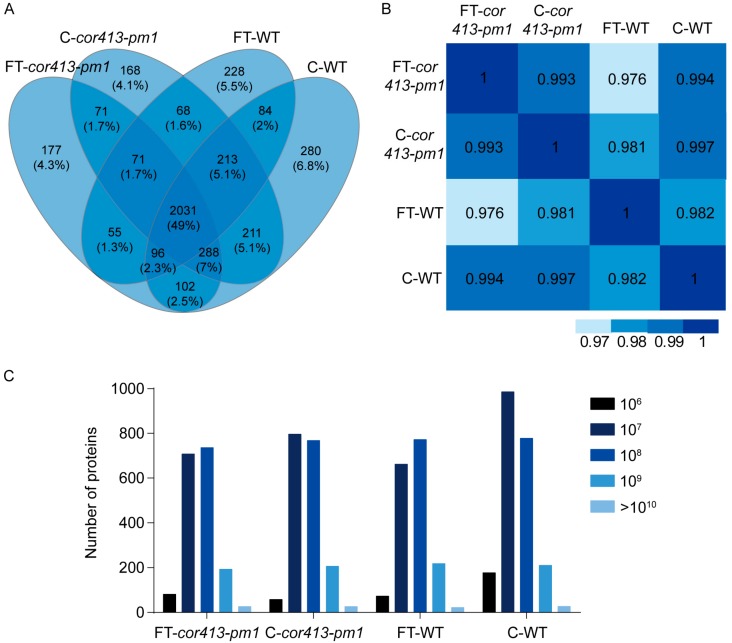
Analysis of qualitative and quantitative identification of proteins of *cor413-pm1* and WT seedling shoots. (**A**) Venn diagram of qualitatively identified proteins in *cor413-pm1* and WT when plants were exposed or nonexposed to a freezing treatment. C-*cor413-pm1*: *cor413-pm1* seedling shoots not exposed to freezing temperature; FT-*cor413-pm1*: *cor413-pm1* seedling shoots exposed to freezing temperature; C-WT: WT seedling shoots not exposed to freezing temperature; FT-WT: WT seedling shoots exposed to freezing temperature. The percentage in parentheses show the proportion of this protein group in a total of 4143 proteins. (**B**) Pearson correlation analysis of the four plant-treatment combinations. Abscissa and ordinate indicate the names of treatment groups. Shades of blue color represent correlation coefficients. (**C**) Distribution of numbers of proteins observed along a range of signal intensities in each treatment group.

Fourteen-day-old seedlings grown in soil were sampled for proteomic analysis. The treatment condition of freezing stress was placing pots with seedlings at −8 °C for 1 h. Total proteins in the seedling shoots were extracted from *cor413-pm1* and WT plants exposed to control and freezing treatment conditions, with three replicates for each of the four plant-treatment combinations. A label-free mass-spectrometry-based proteomics approach was used to explore the DAPs between treatment groups. The separation of chromatographic peaks was shown in Total Ions Chromatograms (TIC) (Supplementary Figure S2). A Venn diagram shows the distribution of a total of 4143 qualitative proteins in *cor413-pm1* and WT of the control and freezing treatment groups (Figure 2A). The types of proteins identified in the four groups were highly dependent on plant type and treatment, but about 49% of the total identified proteins were simultaneously detected in all groups. Figure 2A also showed that a total of 2743 common proteins were identified both in control condition cultured *cor413-pm1* and WT, while 2253 common proteins were identified in both freezing-treated *cor413-pm1* and WT. More differences were observed between *cor413-pm1* and WT after freezing treatment (Figure 2A). A Pearson correlation analysis of four groups indicated that the correlation coefficients of quantitative proteins among the four treatment groups were above 0.9 (Figure 2B). The correlation coefficient between *cor413-pm1* and WT was 0.997 under normal conditions, whereas the correlation coefficient between *cor413-pm1* and WT was lower (0.976) after exposure to the freezing treatment (Figure 2B). These data suggest that the protein expression profile of the mutant *cor413-pm1* was similar to that of the WT under normal conditions, whereas the protein expression profiles differed after exposure to the freezing treatment. In addition, the distribution of numbers of different proteins observed in a log scale of signal intensities (which represents the abundances of each protein) mainly concentrated in the 10^7^–10^8^ range in all four treatment combinations (Figure 2C). Further, when exposed to the freezing treatment, the number of low abundance (10^6^–10^7^) proteins in *cor413-pm1* mutant (FT-*cor413-pm1*) was more than WT (FT-WT) (Figure 2C).

**Figure 3:**
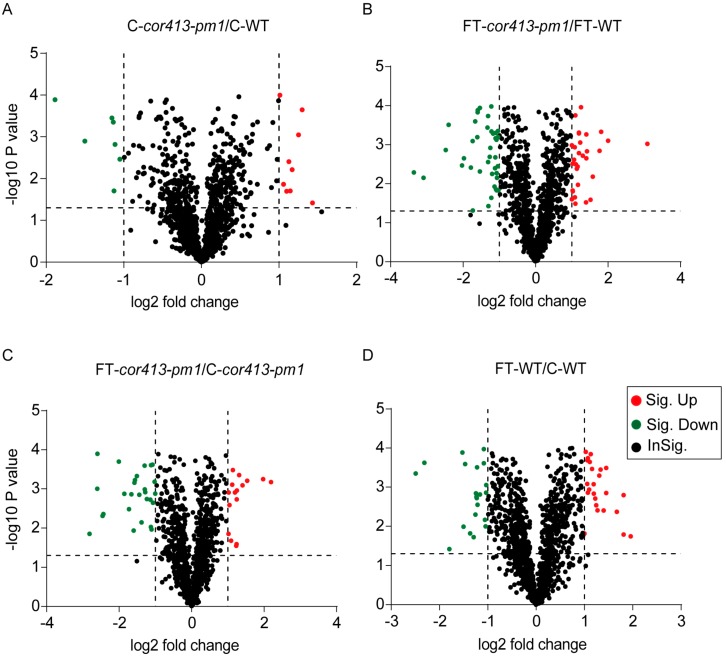
Volcano plots depicting the variance in expression between *cor413-pm1* and WT samples. The significantly changed proteins in (**A**) C-*cor413-pm1* and C-WT, (**B**) FT-*cor413-pm1* and FT-WT, (**C**) FT-*cor413-pm1* and C-*cor413-pm1*, and (**D**) FT-WT and C-WT. Dotted vertical lines indicate standards deviation from the mean fold change and *p*-value. Points plotted in the upper right and upper left corners have the highest precision and greatest absolute fold change. Red: significantly upregulated DAPs; green: significantly downregulated DAPs.

Among all identified proteins, a total of 3139 proteins were accurately quantitated. For screening of DAPs, the signal intensity of a protein in a given sample was compared to the signal intensity of the control. When the ratio was greater than twofold or less than half, the protein was considered to be an upregulated or downregulated DAP, respectively. The fold changes and *p*-values of DAPs are listed in Supplementary Table S1. The volcano plot exhibits high similarity between *cor413-pm1* and WT without freezing treatment (Figure 3A). On the other hand, there was a large difference in amounts of both high and low abundance proteins between *cor413-pm1* and WT after exposure to the freezing treatment compared with that under normal conditions (Figure 3B). It is important to note that many downregulated and upregulated DAPs were also identified in freezing treated and nonfreezing treated *cor413-pm1* or WT (Figure 3C,D).

The numbers of DAPs detected in the comparison of samples are shown in Table 1. A total of 1125, 923, 946, and 1346 DAPs (upregulated plus downregulated DAPs) was detected in the comparison of FT-*cor413-pm1* vs. FT-WT, C-*cor413-pm1* vs. C-WT, FT-*cor413-pm1* vs. C-*cor413-pm1*, and FT-WT vs. C-WT, respectively (Table 1). The number of upregulated proteins was similar to downregulated proteins in FT-*cor413-pm1* vs. FT-WT, but the number of upregulated proteins was lower than that of the downregulated proteins in C-*cor413-pm1* vs. C-WT (Table 1). The ratio of upregulated to downregulated proteins in FT-*cor413-pm1* vs. C-*cor413-pm1* (1:1.3) was higher than FT-WT vs. C-WT (1:1.9) (Table 1). The number of upregulated and downregulated proteins in *cor413-pm1* across treatments (e plus g in Table 1, 946 proteins) was lower than the number in the WT (f plus h in Table 1, 1346), which demonstrated that the response to freezing treatment in *cor413-pm1* was weaker than in WT.

**Figure 4:**
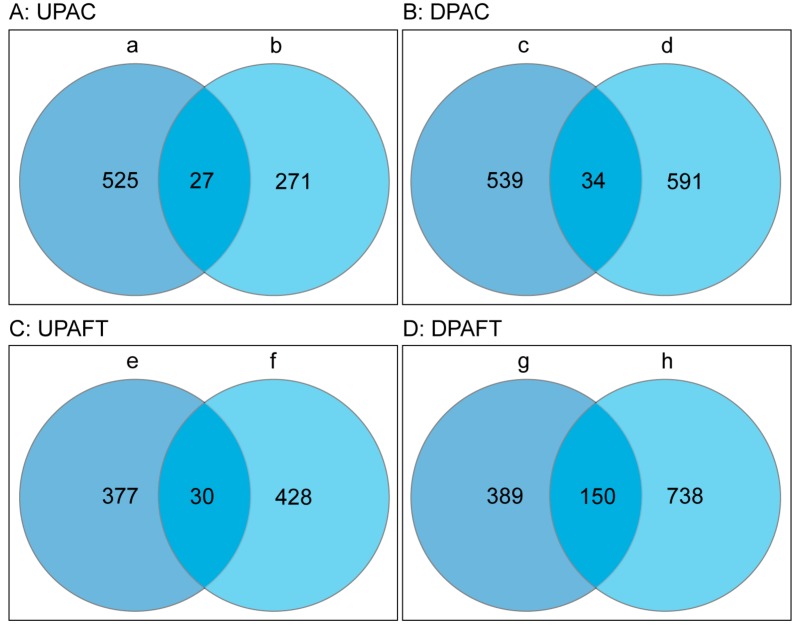
Venn diagrams of DAPs. (**A**) Upregulated proteins and (**B**) downregulated proteins in *cor413-pm1* compared with WT at both freezing and normal temperatures. (**C**) Upregulated proteins and (**D**) downregulated proteins of the mutant *cor413-pm1* and WT after exposure to freezing temperature. The total numbers of proteins are shown for each group. a: the upregulated proteins of FT-*cor413-pm1* vs. FT-WT; b: the upregulated proteins of C-*cor413-pm1* vs. C-WT; c: the downregulated proteins of FT-*cor413-pm1* vs. FT-WT; d: the downregulated proteins of C-*cor413-pm1* vs. C-WT; e: the upregulated proteins of FT-*cor413-pm1* vs. C-*cor413-pm1*; f: the upregulated proteins of FT-WT vs. C-WT; g: the downregulated proteins of FT-*cor413-pm1* vs. C-*cor413-pm1*; h: the downregulated proteins of FT-WT vs. C-WT. UPAC: upregulated proteins associated with *COR413-PM1*, DPAC: downregulated proteins associated with *COR413-PM1*, UPAFT: upregulated proteins associated with freezing treatment, DPAFT: downregulated proteins associated with freezing treatment.

We found 61 (1.94%) DAPs associated with *COR413-PM1*. Among them, a total of 27 upregulated proteins occurred in both FT-*cor413-pm1* vs. FT-WT group (a) and C-*cor413-pm1* vs. C-WT group (b) (Table 1, Figure 4A). A total of 34 downregulated proteins were found in both the FT-*cor413-pm1* vs. FT-WT group (c) and C-*cor413-pm1* vs. C-WT group (d) (Table 1; Figure 4B). The 180 (5.73%) DAPs associated with the freezing treatment in both the mutant *cor413-pm1* and WT were selected for further analysis. Venn diagrams illustrate the shared 30 DAPs of upregulated proteins (Figure 4C) and shared 150 DAPs of downregulated proteins (Figure 4D) associated with freezing treatment. The details of the upregulated and downregulated proteins are listed in Supplementary Table S2.

**Figure 5:**
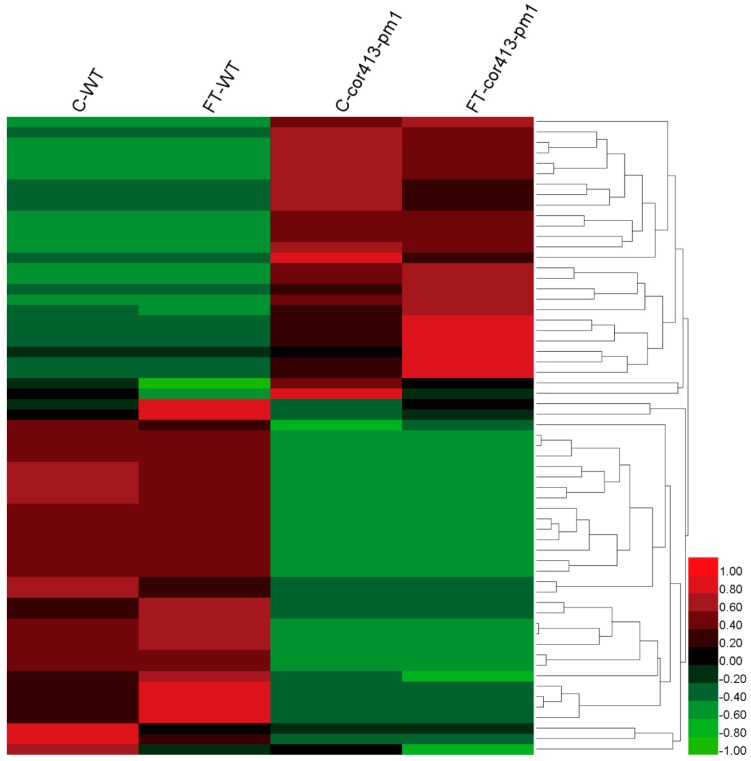
Heatmap based on intensities of the 61 DAPs associated with *COR413-PM1*. Red color indicates the higher protein intensities which set “1”, black color indicates “0”, and green color indicates lower intensities which set “−1” of C-WT, FT-WT, C-*cor413-pm1*, and FT-*cor413-pm1*. Lines show the clustering of the DAPs in the heatmap.

**Figure 6:**
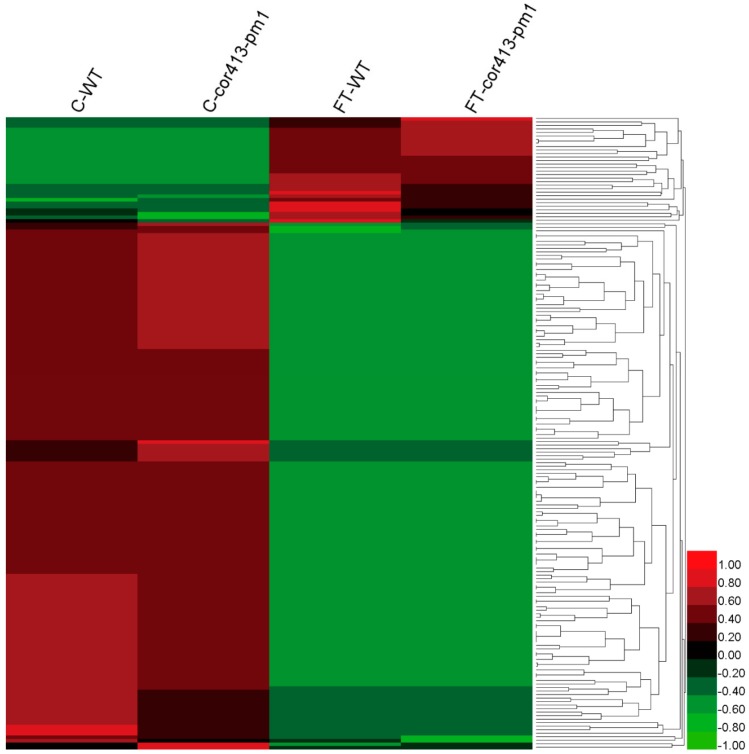
Heatmap based on intensities of the 180 DAPs associated with freezing treatment.

In total, intensities of 61 DAPs were larger different between *cor413-pm1* and WT (Figure 5) and 180 DAPs were significantly larger different between the freezing treatment and normal treatment (Figure 6), which suggests that 61 DAPs and 180 DAPs were part of a specific response to *COR413-PM1* and the freezing treatment, respectively.

### 2.3. Categorization of DAPs in Seedling Shoots

**Figure 7:**
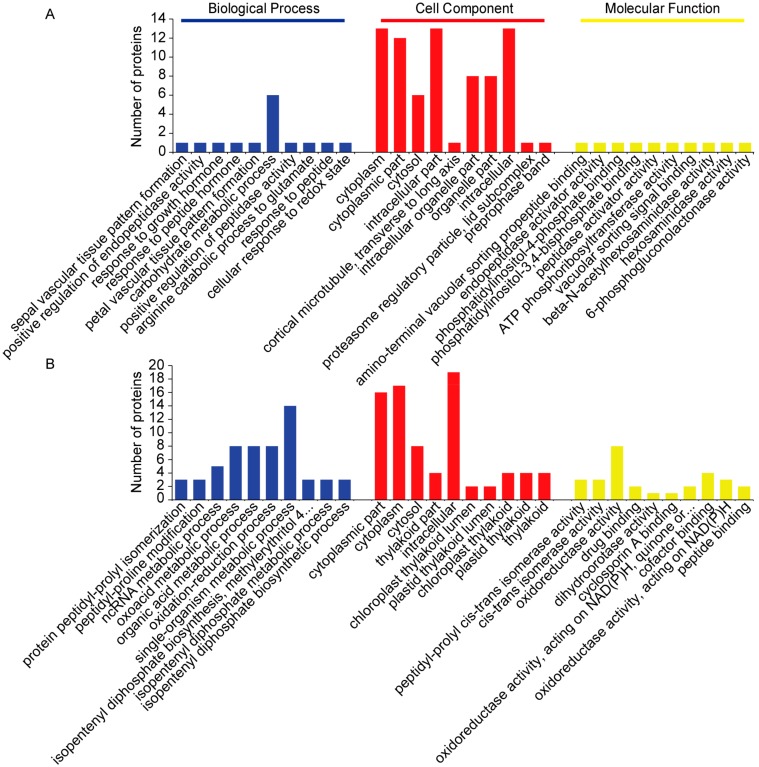
GO analysis of the DAPs associated with cold-regulated multispanning transmembrane gene *COR413-PM1*. The top 10 terms significantly enriched in the three categories (biological process (BP), cellular component (CC), and molecular function (MF)) of the GO terms analysis are displayed. (**A**) GO analysis of 27 upregulated DAPs associated with *COR413-PM1* in seedling shoots; (**B**) GO analysis of 34 downregulated DAPs associated with *COR413-PM1* in seedling shoots. Terms in each category are arranged, from left to right, from the highest to lowest *p*-values (*p* < 0.05). The numbers of proteins are shown on the y-axis.

**Figure 8:**
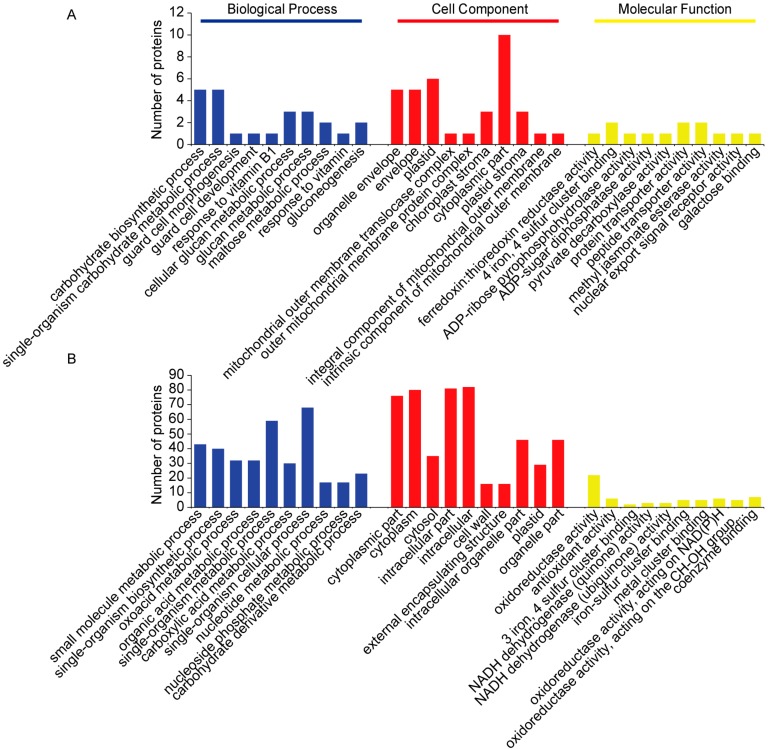
GO analysis of the DAPs associated with freezing treatment. The top 10 terms significantly enriched in the three categories (BP, CC, and MF) of the GO terms analysis are displayed. (**A**) GO analysis of 30 upregulated DAPs associated with freezing treatment; (**B**) GO analysis of 150 downregulated DAPs associated with freezing treatment.

To gain insight into the functional categories of DAPs that were altered between *cor413-pm1* and WT seedling shoots, Gene Ontology (GO) categories were assigned based on the Gene Ontology database (Available online: http://www.geneontology.org/). The distribution charts of the GO terms and pathway enrichment according to the corresponding classifications of biological process (BP), cellular component (CC), and molecular function (MF) are shown in Figure 7 and Figure 8. These figures give an overview of the GO analysis with up to 10 significantly enriched terms in the BP, CC, and MF categories. The majority of the 27 upregulated DAPs found in *cor413-pm1* seedling shoots appeared to be related to 447 biological changes, with 105 terms significantly enriched in BP. A total of 115 GO terms were enriched in CC, of which 35 terms reached statistical significance. Thirty-nine terms were significantly enriched in MF (Figure 7A). The majority of the 34 downregulated DAPs found in *cor413-pm1* seedling shoots appeared to be related to 580 BP terms, of which 157 terms were significantly enriched. A total of 75 terms were enriched in CC, of which 30 terms were significantly enriched. A total of 38 terms were significantly enriched in MF in this dataset (Figure 7B). The top 10 terms significantly enriched in the three categories are shown in Figure 7. The first three BP terms of the 27 upregulated proteins were sepal vascular tissue pattern formation, positive regulation of endopeptidase activity, and response to growth hormone (Figure 7A). The 34 downregulated proteins’ first three BP terms were protein peptidyl-prolyl isomerization, peptidyl-proline modification, and ncRNA metabolic process (Figure 7B). The first three CC terms of the 27 upregulated proteins were cytoplasm, cytoplasmic part, and cytosol (Figure 7A). The 34 downregulated proteins’ first three CC terms were mainly related to cytoplasmic part, cytoplasm, and cytosol (Figure 7B). The first three MF terms of the 27 upregulated proteins were amino-terminal vacuolar sorting propeptide binding, endopeptidase activator activity, and phosphatidylinositol-4-phosphate binding (Figure 7A). The 34 downregulated proteins’ first three MF terms were mainly related to peptidyl-prolyl cis-trans isomerase activity, cis-trans isomerase activity, and oxidoreductase activity (Figure 7B).

The majority of the 30 upregulated DAPs in the seedling shoots that associated with freezing treatment appeared to be related to 429 biological changes, with 92 terms significantly enriched in BP. A total of 95 GO terms were enriched in CC, of which 29 terms reached statistical significance. Forty-three terms were significantly enriched in MF (Figure 8A). The majority of the 150 downregulated DAPs associated with freezing treatment appeared to be related to 1258 BP terms, of which 570 terms were significantly enriched. A total of 195 terms were enriched in CC, of which 100 terms were significantly enriched. One hundred and forty terms were significantly enriched in MF (Figure 8B). The top 10 terms significantly enriched in the three categories are shown in Figure 8. The first three BP terms of the 30 upregulated proteins were carbohydrate biosynthetic process, single-organism carbohydrate metabolic process, and guard cell morphogenesis (Figure 8A). The 150 downregulated proteins’ first three BP terms were mainly related to small molecule metabolic, single-organism biosynthetic, and oxoacid metabolic processes (Figure 8B). The first three CC terms of the 30 upregulated proteins were organelle envelope, envelope, and plastid (Figure 8A). The 150 downregulated proteins’ first three CC terms were same as the 34 downregulated proteins (Figure 8B). The first three MF terms of the 30 upregulated proteins were ferredoxin: thioredoxin reductase activity, 4 iron and 4 sulfur cluster binding, and ADP-ribose pyrophosphohydrolase activity (Figure 8A). The 150 downregulated proteins’ first three MF terms were mainly related to oxidoreductase activity, antioxidant activity, and 3 iron, 4 sulfur cluster binding (Figure 8B).

**Figure 9:**
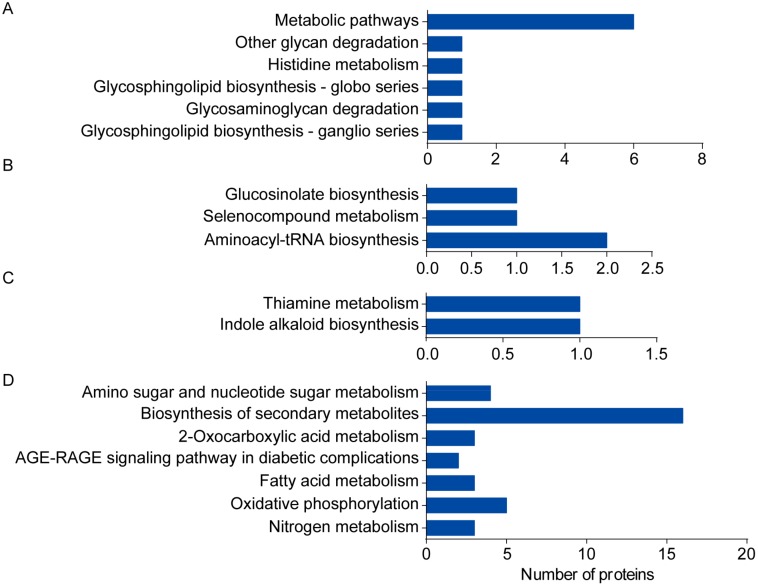
KEGG pathway enrichment of the DAPs associated with cold-regulated multispanning transmembrane gene *COR413-PM1* and freezing treatment in seedling shoot material. (**A**) KEGG pathway enrichment of 27 upregulated DAPs associated with *COR413-PM1* gene; (**B**) KEGG pathway enrichment of 34 downregulated DAPs associated with *COR413-PM1* gene; (**C**) KEGG pathway enrichment of 30 upregulated DAPs associated with freezing treatment; (**D**) KEGG pathway enrichment of 150 downregulated DAPs associated with freezing treatment.

Analysis results from using the Kyoto Encyclopedia of Genes and Genomes (KEGG) Pathway database (Available online: http://www.kegg.jp/) indicated that the 27 upregulated DAPs found in *cor413-pm1* seedling shoots mapped to 21 KEGG pathways. These most significantly enriched KEGG pathways were involved in glycosphingolipid biosynthesis-ganglio series (ath00604, *p* = 8.30 × 10^−3^), glycosaminoglycan degradation (ath00531, *p* = 1.16 × 10^−2^), glycosphingolipid biosynthesis-globo series (ath00603, *p* = 1.49 × 10^−2^), histidine metabolism (ath00340, *p* = 2.96 × 10^−2^), other glycan degradation (ath00511, *p* = 2.96 × 10^−2^), and metabolic pathways (ath01100, *p* = 4.76 × 10^−2^) (Figure 9A). The KEGG pathways of the 34 significantly downregulated DAPs in *cor413-pm1* functioned in aminoacyl-tRNA biosynthesis (ath00970, *p* = 1.83 × 10^−2^), selenocompound metabolism (ath00450, *p* = 3.32 × 10^−2^), and glucosinolate biosynthesis (ath00966, *p* = 3.51 × 10^−2^) (Figure 9B). Panels of upregulated and downregulated DAPs associated with *COR413-PM1* of KEGG pathways are shown in Supplementary Figure S3.

Moreover, the analysis with the KEGG Pathway database indicated that the 30 upregulated DAPs associated with freezing treatment of the seedling shoots mapped to 11 KEGG pathways. The most significantly enriched KEGG pathways were involved in indole alkaloid biosynthesis (ath00901, *p* = 7.47 × 10^−3^) and thiamine metabolism (ath00730, *p* = 1.37 × 10^−2^) (Figure 9C). The KEGG pathways of the 150 significantly downregulated DAPs were associated with freezing treatment and nitrogen metabolism (ath00910, *p* = 7.71 × 10^−3^), oxidative phosphorylation (ath00190, *p* = 2.00 × 10^−2^), fatty acid metabolism (ath01212, *p* = 3.04 × 10^−2^), AGE-RAGE signaling pathway in diabetic complications (ath04933, *p* = 3.22 × 10^−2^), 2-oxocarboxylic acid metabolism (ath01210, *p* = 3.51 × 10^−2^), biosynthesis of secondary metabolites (ath01110, *p* = 4.14 × 10^−2^), and amino sugar and nucleotide sugar metabolism (ath00520, *p* = 4.19 × 10^−2^) (Figure 9D). Panels of upregulated and downregulated DAPs associated with freezing treatment of KEGG pathways are shown in Supplementary Figure S4. The main proteins of the DAPs associated with *COR413-PM1* and freezing treatment are shown in Supplementary Table S2.

### 2.4. qRT-PCR Analysis

Among DAPs, we selected eight proteins that have been reported in relation to low-temperature stress, and qRT-PCR validation of these proteins was completed. Among them, it was demonstrated in this study that fatty acid biosynthesis 1 (FAB1)/beta-ketoacyl-ACP synthetase 2 (KAS2) (AT1G74960), GLN phosphoribosyl pyrophosphate amidotransferase 2 (ASE2) (AT4G34740), and SM-like protein 4 (LSM4) (AT5G27720) were associated with the freezing treatment. The other five proteins are vitamin C defective 1 (VTC1)/cytokinesis defective 1 (CYT1)/GDP-mannose pyrophosphorylase (GMP1) (AT2G39770), trehalose-6-phosphatase synthase S7 (TPSA/TPS7) (AT1G06410), fructokinase 3 (FRK3/FRK6) (AT1G66430), sucrose phosphate synthase A1 (SPSA1) (AT5G20280), and pyrimidine 1 (PYD1) (AT3G17810).

**Figure 10:**
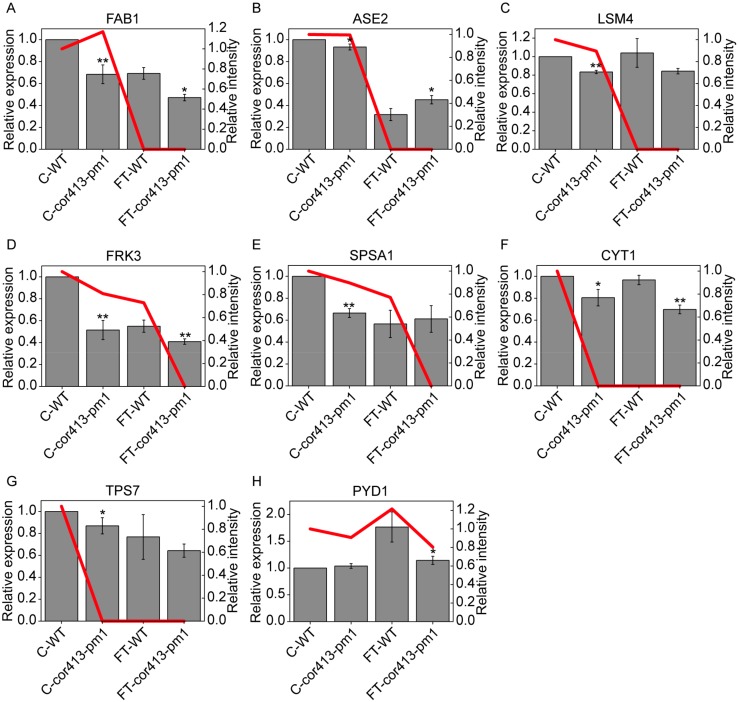
Proteomics targeting intensities (line chart) and quantitative real-time PCR (qRT-PCR) (histogram) of candidate DAPs. Expression levels of (**A**) fatty acid biosynthesis 1 (FAB1)/beta-ketoacyl-acp synthetase 2 (KAS2), (**B**) GLN phosphoribosyl pyrophosphate amidotransferase 2 (ASE2), (**C**) SM-like protein 4 (LSM4), (**D**) fructokinase 3 (FRK3/FRK6), (**E**) sucrose phosphate synthase A1 (SPSA1), (**F**) vitamin C defective 1 (VTC1)/cytokinesis defective 1 (CYT1), (**G**) trehalose-6-phosphatase synthase S7 (TPSA/TPS7), and (**H**) pyrimidine 1 (PYD1) in the seedling shoots of WT and *cor413-pm1*. Red lines are proteomics targeting intensities. Data shown are means ± SD (*n* = 4). The analysis for significant differences is the same treatment condition group. * *p* < 0.05, ** *p* < 0.01 (Student’s *t*-test).

Expression levels of most of the eight proteins were downregulated in *cor413-pm1* when compared to those of WT, whether under normal or freezing temperatures. The expression levels of *FAB1*, *ASE2*, *FRK3*, *SPSA1*, and *PYD1* were consistent with protein intensities (Figure 10A,B,D,E,H). The expression levels of *LSM4*, *CYT1*, and *TPS7* were not consistent with their protein intensities (Figure 10C,F,G). Previous studies have shown that gene expression level might not be consistent with protein level because of post-translational regulation mechanisms [32,33,34]. The intensities of FAB1, ASE2, LSM4, CYT1, and TPS7 in freezing treatment were not consistently detectable in both plant lines (Figure 10A–C,F,G) and those proteins might be present but intensities were too weak to detect using the proteomic method.

### 2.5. Protein–Protein Interaction Analysis of DAPs

**Figure 11:**
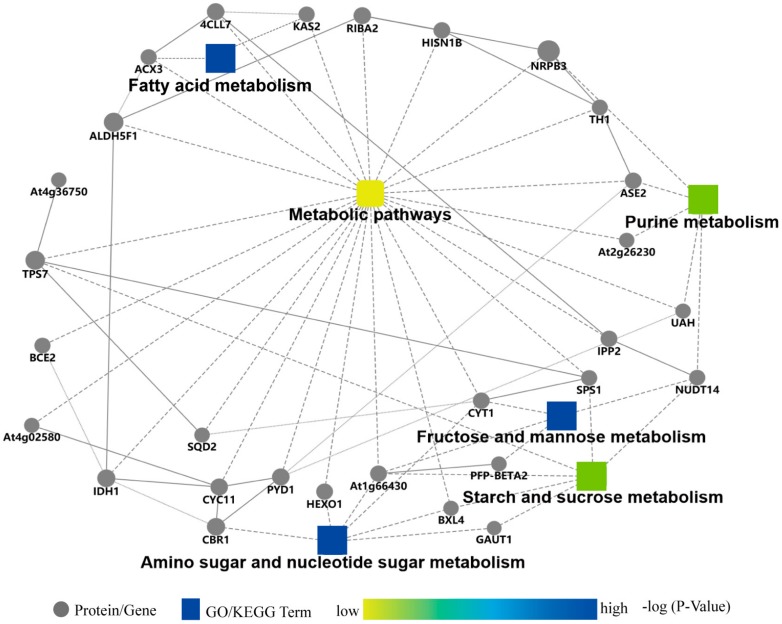
The protein–protein interaction (PPI) network of the main DAPs involved in metabolic pathways. FAB1 (KAS2) enriches in the fatty acid biosynthesis and metabolism pathway. FRK3 (AT1G66430) and CYT1 participate in more than one sugar metabolic pathway. TPS7 and SPSA1 enrich in starch and sucrose metabolism. ASE2 participates in the purine metabolic pathway.

To predict the relationships among the selected eight DAPs in the different KEGG pathways, protein–protein interaction (PPI) networks were generated by the webtool Search Tool for the Retrieval of Interacting Genes (STRING) [35] and TAIR10. Several KEGG pathways were enriched in the PPI network, including the metabolisms of amino and nucleotide sugars, fructose and mannose, starch and sucrose, and purine. We ultimately obtained 29 related proteins participating in PPI networks (Figure 11). Among pathways, we found that, confirmed using qRT-PCR, four DAPs, including FAB1/KAS2, FRK3/FRK6 (AT1G66430), SPSA1/SPS1, and ASE2, were involved in metabolic processes of fatty acids, sugars, and purine, which suggests that *COR413-PM1* mainly regulated freezing tolerance through affecting these metabolism processes in plants.

## 3. Discussion

Plants regulate growth through metabolism and biosynthesis to acclimate to a changing external environment. The changes to structure and function of the cell membrane due to low temperatures result in an increase in membrane permeability, electrolyte extravasation, and relative electrical conductivity [30]. Membrane lipid peroxidation often occurs in plants under adverse conditions [36]. The content of the membrane lipid peroxidation product MDA can reflect the extent of cell membrane lipid peroxidation and the degree of damage to cells [31]. The membrane protein content of *alfalf* increased after cold acclimation for 2–3 days at 4 °C [37]. A similar functioning protein in wheat, *WPI6*, is also a plasma membrane protein with two predicted membrane-spanning domains that play a protective role in maintaining plasma membrane function during cold acclimation [38]. These studies indicate that the stability of plant membrane proteins is very important for cold stress tolerance in plants.

Results from the qRT-PCR implied that the expression of genes were diminished in the mutant *cor413-pm1*, which confirmed the results of the proteomic analysis and suggested that proteins might be implicated in increasing plant tolerance to cold. Stability of the plasma membrane during freezing-induced dehydration is affected by many factors associated with endomembranes and cytoplasm, such as the chloroplast envelope lipid composition and accumulation of sugars [39]. FAB1 participates in fatty acid biosynthesis and metabolism (Figure 11 and Supplementary Table S2). Cell membrane fluidity is closely related to cold tolerance in plants [40]. An increase in unsaturated fatty acids can prevent lipid solidification at low temperatures [41]. The desaturation of fatty acids during chilling acclimation is one of the factors involved in conferring low-temperature tolerance in young tobacco leaves [41]. Plants with a high proportion of *cis*-unsaturated fatty acids are resistant to chilling, which has been demonstrated in spinach (*Spinacia oleracea* L.) and *Arabidopsis* [42]. Added oxidative stress leads to a decrease in membrane fluidity through an increase of free radicals [43]. The expression of *FAB1* was significantly lower in the mutant *cor413-pm1*, whether under normal conditions or subjected to freezing stress (Figure 10A). The mutant *fab1* appeared to be susceptible to cellular damage caused by low temperatures (2–6 °C) for long periods. The stress affected photosynthesis in *fab1* by the degradation of its chloroplasts and consequently resulted in plant death. [44]. The mutant *fab1* had increased levels of 16:0 fatty acids and concomitantly decreased levels of 18:0 fatty acids likely due to a mutation in the *KAS2* (*FAB1*) gene, which caused a structural instability of the gene product during fatty acid synthesis [45,46]. Content of phosphatidylglycerol, associated with chilling stress, decreased by 10% in the mutant *fab1* leaves compared with that of the WT *Arabidopsis* leaves [47]. Those results show that different fatty acid compositions, such as the ratio between saturated and unsaturated fatty acids, play key roles in acclimation to ambient temperatures. *COR413-PM1* might affect the membrane structure through regulation of FAB1, which further determines the tolerance to freezing stress in *Arabidopsis*.

Sucrose is important for the maintenance of osmotic pressure in cellular metabolism as a signal molecule and in altering cell wall extensibility [48]. Fructose is a breakdown product of sucrose either by invertases or sucrose synthases [49]. Intensities of FRK3, SPSA1, CYT1, and TPS7 decreased due to the freezing treatment of *cor413-pm1* and WT seedlings when compared to those of the respective seedlings of the control groups (Figure 10D–G). FRK3 (AT1G66430) and CYT1 participate in more than one kind of sugar metabolism (Figure 11 and Supplementary Table S2). TPS7 and SPSA1 enriches in starch and sucrose metabolism (Figure 11 and Supplementary Table S2). The FRK3/FRK6 gene, one of the seven main fructose-phosphorylating enzymes, is a single plastid-localized FRK [49]. Gas chromatography-mass spectrometry (GC-MS) was used to analyze the primary metabolic profile of the double-mutants of *AtFRK6* and *AtFRK7* seeds and revealed that TCA cycle organic acids and fatty acid metabolism decreased in these mutants [50]. The reduction of the UDP-glucose pool was observed by RNAi targeting of *FRK2* in a hybrid aspen and resulted in thinner fibers and a lower proportion of cellulose in cell walls [51]. *CYT1* is a key regulatory target in ascorbic acid (AsA) biosynthesis [52]. *CYT1* encodes a GDP-mannose pyrophosphorylase which provides GDP-mannose, a principal component of cell wall carbohydrate biosynthesis. The *CYT1* promoter was found to contain two DRE core sequences (ACCGAC) upstream of the transcription start site. *Arabidopsis ethylene response factor 98* (*AtERF98*) can specifically bind to the DRE-2 fragment and acts as a positive regulator of *CYT1* at the gene expression level [53]. The expression of the *TPS* gene in winter wheat subjected to various low-temperature treatments was measured with the Solexa sequencing platform, and a cluster analysis found that significantly higher gene expression was observed in varieties with strong tolerance than those with weak tolerance to low-temperature treatments. Furthermore, the *TPS* gene in wheat was involved in starch and sugar metabolism [54]. *SPSA1* is one of the four SPS genes encoding sucrose-phosphate synthase (SPS) enzymes. Functional sucrose biosynthesis at low temperatures in overexpressing SPS plants reduced the inhibition of photosynthesis and increased the rate at which freezing tolerance developed [55]. Those results suggest that FRK3, CYT1, TPS7, and SPSA1 might be involved in tolerance to freezing stress in the mutant *cor413-pm1* and can affect cell wall extensibility through sugar metabolism.

It was shown that ASE2 participated in the purine metabolism pathway (Figure 11 and Supplementary Table S2). Purine nucleotides are directly involved in the synthesis of nucleic acids which serve as energy sources for plant growth [56]. The isozymes are encoded by the three genes *ASE1* (AT2G16570), *ASE2*, and *ASE3* (AT4G38880). Both *ASE1* and *ASE2* were localized to chloroplasts [57]. *ASE2*, one of the three ATase isozymes responsible for the first committed step of de novo purine biosynthesis, is not only important for cell division but also for chloroplast biogenesis [57]. There are direct interactions between the COR gene expression originating from a cold-induced signal transduction pathway and the redox state of the chloroplast [58,59]. *ASE2* transgenic tobacco plants as well as *Arabidopsis* mutants exhibit strong growth retardation and severe chlorosis in leaves [60]. These results support the role of ASE2 in tolerance to freezing stress through purine metabolism.

The tolerance to freezing stress in the mutant *cor413-pm1* may be also influenced by abiotic stress and hormone signal transduction. It was reported that *LSM4* genes were related to abscisic acid and osmotic stress signaling [61]. The loss of *LSM4* lead to the growth retardation and greater sensitivity to salt. LSM4 was enriched in RNA degradation pathway. *PYD1* initiated the degradation of uracil or thymine nucleobases [62]. The *A. thaliana* mutant *pyd1* exhibited delayed germination and seedling development and decreased cytosolic invertase activity that lead to an accumulation of sucrose [62]. The researchers assumed the interference of *PYD1* with ABA signaling by the fact of ABA-responsive genes having been deregulated in *pyd1* mutants and an increased *PYD1* expression in wild type seedlings upon ABA treatment [62].

## 4. Materials and Methods

### 4.1. Plant Materials and Growth Conditions

Wild-type (WT) seeds of *Arabidopsis* ecotype Columbia-0 (Col-0) were preserved in the Key Laboratory of Biology and Genetic Improvement of *Triticeae* Crops, Ministry of Agriculture of China. Transfer DNA insertion lines of the mutant *cor413-pm1* (SALK_014871, *COR413-PM1*, AT2G15970) were obtained from the *Arabidopsis* Biological Resource Center (ABRC) (Available online: http://www.Arabidopsis.org). Loss-of-function of the mutant *cor413-pm1* was confirmed by RT-PCR analysis. After WT and *cor413-pm1* seeds were subjected to surface disinfection, they were sown on MS_0_ solid medium, refrigerated for 2–3 days at 4 °C, and then grown for 10 days in a culture chamber (22 °C, relative humidity 65%, photoperiod 16 h/8 h). Seedlings were then transplanted to 140-cm diameter by 120-cm height pots with fertilized soil (vermiculite 2:1) and grown in a greenhouse (22 °C, 16 h/8 h light/dark cycle). After 14 days, at least four pots of each WT and *cor413-pm1* seedlings were moved into a refrigerator to expose seedlings to a −8 °C freezing treatment for 1 h. Four pots of each plant line, WT and *cor413-pm1*, grown at room temperature were used as control groups.

### 4.2. Relative Electrical Conductivity

Leaves of WT and *cor413-pm1* seedlings (0.03 g) of both the control and freezing treatments were collected into 15 mL tubes containing 5 mL deionized water. They were vacuumed for 30 min with a CentriVap console (Labconco, Kansas City, MO, USA) and then sat for 45 min at room temperature. The electrical conductivity (EC) of each sample was measured as S_1_, and deionized water without leaves was measured as S_0_. All samples were boiled for 10 min and then reduced to room temperature, and the final EC was measured as S_2_. The relative electrical conductivity was calculated as follows: REC = (S_1_ − S_0_)/(S_2_ − S_0_) [30].

### 4.3. Protein Extraction

The filter-aided sample preparation method (FASP) [63] was used to prepare the samples. After exposure to a −8 °C freezing treatment for 1 h and a recovery period for 8 h at 22 °C, 50 mg of *Arabidopsis* seedling shoot samples were placed in a 2-mL centrifuge tube with a steel ball and then added to liquid nitrogen for subsequent protein extraction. Cells in each sample were effectively pulverized in liquid nitrogen by using a Cell Disruption System (Retsch, Haan, Germany) for 1 min with a frequency of 20 times/s. They were then incubated with 400 μL extraction buffer (8 M Urea, 2 mM EDTA, 20 mM CaCl_2_, 200 mM NaCl, 100 mM Tris-HCl, pH 8.1) and freeze-thawed 3–5 times. The crude extract was centrifuged at 13,000× *g* for 20 min at 4 °C. The clear supernatant was loaded onto a filtration device (Amicon Ultra 0.5 mL Centrifugal Filters, 10 K, Millipore, Burlington, MA, USA) and then centrifuged at 13,000× *g* for 25 min at 4 °C. The filtrate was poured out from the outer tube. The filtration device was incubated for 60 min at 30 °C on a shaker after 200 μL DTT buffer (50 mM Dithiothreitol, 8 M Urea, 100 mM Tris-HCl, pH 8.1) was added in the inner tube for reduction of the disulfide bonds in the protein extracts. The incubated filtration device was centrifuged at 13,000× *g* for 30 min and then the filtrate was removed. The concentrate was diluted with 200 μL UA buffer (8 M Urea, 100 mM Tris-HCl, pH 8.1) and centrifuged three times to replace the DTT in each protein sample. Subsequently, freshly prepared IAA buffer (50 mM Iodoacetamide, 8 M Urea, 100 mM Tris-HCl, pH 8.1) was added and then samples were incubated for 30 min at 30 °C in the dark. Then, the samples were centrifuged at 13,000× *g* for 30 min. After three buffer exchanges with 8 M Urea, the resulting concentrate was diluted two times with 100 μL 50 mM NH_4_HCO_3_ solution. Finally, 50 µg protein extract was digested with 0.5 µg Trypsin (Promega, Mannheim, Germany) at 1:100 enzyme/protein concentration for 10 h at 37 °C. The enzyme cut samples were centrifuged at 13,000× *g* for 30 min with filtration devices. Ultrafiltration of the peptide mixture was collected and quantified based on the OD 280 values. The samples were directly injected for mass spectrometry analysis. Three biological replications were prepared in the study.

### 4.4. Mass Spectrometric Analysis

A label-free quantification (LFQ) mass spectrometry (MS) method was used to profile the *Arabidopsis* seedling shoot materials. For each run, 1 μL peptides were loaded onto an Easy-nano1000 liquid chromatography (LC) system (Thermo Fisher Scientific, San Jose, CA, USA) equipped with a C18 PepMap trap precolumn (100 μm × 20 mm, Thermo Fisher Scientific). The eluted peptides were separated with a linear acetonitrile gradient (3–90% over 90 min) in 0.1% formic acid at a flow rate of 200 nL/min on a C18 Tip column (75 μm × 150 mm, Thermo Fisher Scientific, San Jose, CA, USA) with a spray voltage of 2.3 kV. The peptide ions in the spray were analyzed in data-dependent acquisition mode on a Q-Excative Plus Orbitrap MS (Thermo Fisher Scientific, San Jose, CA, USA). The main parameter settings for the MS analysis are shown in Table 2.

### 4.5. Protein Identification and Quantitation

The qualitative analysis of identified proteins was performed with the Proteome Discoverer 2.1 software (PD, Thermo Fisher, Foster City, CA, USA). We conducted a plant genome search through the *Arabidopsis* Database (Available online: https://phytozome.jgi.doe.gov) using version Athaliana_167_TAIR10.protein. The qualitative search parameters were as follows: the confidence level of peptides was 6–144 amino acids in length and a maximum of two missed cleavages were allowed. The mass deviation of the parent ion was ±10 ppm and the mass deviation of the fragment ion was 0.02 Da. We used the iodoacetamide of cysteine (Cys) (carbamidomethyl/+57.021 Da) to fix the modification, with the variable modification by methionine (Met) oxidation (oxidation/+15.995 Da) and *N*-acetylation (Acetyl/+42.011 Da). The false discovery rate of peptide search was set to 1%. Three replicates were retrieved together to obtain qualitative results for each plant group.

Maxquant version 1.3.0.5 software (Available online: http://www.coxdocs.org) was used to conduct the quantitative analysis of identified proteins. The original data for each of the three replicates of each treatment group were selected for retrieval in the *Arabidopsis* Database as above. The search standards for the quantitative analysis were the same as those used in the qualitative analysis, such as the mass deviation of the fragment ion, the fixed modification, and the variable modification. Trypsin was used as the default protease and the precursor mass tolerance was ±20 ppm. The minimum length of peptides that could be detected was seven amino acids. The false discovery rate of peptide search was set to 1%. The data information was output in Excel.

Pearson correlation analysis between the quantitation data of the four treatment groups (FT-*cor413-pm1*, C-*cor413-pm1*, FT-WT, C-WT) was performed using SPSS 22.0 data processing software (Available online: https://www.ibm.com/analytics). The degree of linear correlation for the four groups was represented by the absolute value.

### 4.6. Bioinformatics Analysis

The original data were preprocessed and standardized for quality control. They were then screened for reliable proteins based on the phenotypes of the mutant *cor413-pm1* and WT and the intensities of DAPs. The *p*-value threshold was set to 0.05. For screening DAPs, when the ratio of the signal intensity of a protein in a sample was greater than twofold or less than half of the signal intensity of the control sample, the protein was considered to be upregulated or downregulated, respectively. The GO biological functions of DAPs between the WT and the mutant *cor413-pm1* were analyzed by Gene Ontology (Available online: http://www.geneontology.org/). We conducted KEGG Pathway and enrichment analyses of DAPs using the Kyoto Encyclopedia of Genes and Genomes (KEGG) Pathway (Available online: http://www.kegg.jp/). The protein–protein interaction (PPI) analysis was carried out using the Search Tool for the Retrieval of Interacting Genes (STRING) database (Available online: https://string-db.org/cgi/input.pl).

### 4.7. RNA Isolation and Quantitative Real-Time PCR (qRT-PCR)

Total RNA was isolated from *Arabidopsis* using an RNAprep Pure Plant Kit (Tiangen, Beijing, China). Complementary DNA (cDNA) was synthesized using a One-Step gDNA Removal and cDNA Synthesis SuperMix (TransGen Biotech, Beijing, China). The quantitative RT-PCR was implemented using SuperReal PreMix Plus (SYBR Green) (Tiangen, Beijing, China) with an Applied Biosystems ABI 7500 Real-Time PCR System (Thermo Fisher Scientific, San Jose, MA, USA). Each reaction had three replicates.

### 4.8. Accession Codes

The mass spectrometry proteomics data have been deposited to the ProteomeXchange Consortium [64] via the PRIDE [65] partner repository (Available online: http://proteomecentral.proteomexchange.org) with the dataset identifier *PXD010031*.

## 5. Conclusions

The results indicate that the multispanning transmembrane protein COR413-PM1 plays an important role in conferring freezing tolerance in *Arabidopsis*. The mutant *cor413-pm1* showed greater damage to the cellular membrane system under freezing temperatures. The multispanning transmembrane protein COR413-PM1 confirm that the stability of plant membrane proteins is very important for cold stress tolerance in plants. The DAPs are involved in a number of physiological processes and act together to create a new equilibrium when plants are exposed to freezing temperatures. The study of the dynamic changes of proteins in WT and *cor413-pm1* furthers our understanding of the mechanisms of COR413-PM1 at the protein level in response to freezing temperature stress. Three proteins, FAB1, ASE2, and LSM4, were identified and were associated with freezing treatment. The fatty acid metabolism which FAB1 enriched might influence the plasma membrane structure to affect the freezing stress tolerance. Several proteins, including FRK3, SPSA1, CYT1, and TPS7, enriched in sugar metabolism may influence the ability of osmotic adjustment in plants under freezing stress. ASE2 participated in the purine metabolism pathway, which serves as an energy source for plant growth. LSM4 and PYD1 were related to abiotic stress, such as those posed by abscisic acid or salt. Changes in temperature modify the balance of energy absorbed and metabolized in individuals. The interaction between photosynthetic redox, cold acclimation, sugar-signaling pathways, and other processes regulate plant acclimation to low temperatures [59]. Although there are few studies on the membrane localization proteins’ response to low-temperature stress, we firstly indicate that the multispanning transmembrane protein COR413-PM1 plays an important role in the process of low-temperature stress response in plants. The DAPs associated with *COR413-PM1* and freezing treatment were mainly involved in the metabolisms of fatty acids, sugars, and purine. The verification confirmed the proteomic analysis results of four proteins: FAB1, FRK3, SPSA1, and ASE2. As different related proteins were identified, it shows that the complexity of plant signal transduction pathways and freezing tolerance may indicate polygenic controls in *Arabidopsis*.

## Figures and Tables

**Table 1:** Differentially abundant proteins (DAPs) detected in comparison of samples.

Treatment Groups	Upregulated Proteins	Downregulated Proteins	Unchanged Proteins	Total Proteins
FT-*cor413-pm1* ^1^ vs. FT-WT ^2^	552 (a)	573 (c)	1125	2250
C-*cor413-pm1* ^3^ vs. C-WT ^4^	298 (b)	625 (d)	1537	2460
FT-*cor413-pm1* vs. C-*cor413-pm1*	407 (e)	539 (g)	1288	2234
FT-WT vs. C-WT	458 (f)	888 (h)	1243	2589

^1^*cor413-pm1* seedling shoots treated with freezing temperature; ^2^ WT seedling shoots treated with freezing temperature; ^3^ *cor413-pm1* seedling shoots at control temperature; ^4^ WT seedling shoots at control temperature; a: the upregulated proteins of FT-*cor413-pm1* vs. FT-WT; b: the upregulated proteins of C-*cor413-pm1* vs. C-WT; c: the downregulated proteins of FT-*cor413-pm1* vs. FT-WT; d: the downregulated proteins of C-*cor413-pm1* vs. C-WT; e: the upregulated proteins of FT-*cor413-pm1* vs. C-*cor413-pm1*; f: the upregulated proteins of FT-WT vs. C-WT; g: the downregulated proteins of FT-*cor413-pm1* vs. C-*cor413-pm1*; h: the downregulated proteins of FT-WT vs. C-WT.

**Table 2:** Main parameter settings for mass spectrometry (MS).

Name	Resolution Ratio	AGC Target	Maximum IT ^1^	Scan Range	Top N	NCE
Full MS	70,000	3 × 10^6^	50 ms	300–1800 *m*/*z*	/	/
dd-MS2	17,500	1 × 10^5^	45 ms	/	20	27

^1^ injection time.

## Supplementary Materials

Supplementary materials can be found at http://www.mdpi.com/1422-0067/19/9/2572/s1.

## Abbreviations

COR	Cold-regulated
MDA	Malondialdehyde
MS	Mass Spectrometry
DAPs	Differentially Abundant Proteins
GO	Gene Ontology
KEGG	Kyoto Encyclopedia of Genes and Genomes
qRT-PCR	quantitative Real-Time Polymerase Chain Reaction
