# Supplementary material for: Proteomic Analysis of the Function of a Novel Cold-Regulated Multispanning Transmembrane Protein COR413-PM1 in Arabidopsis
Source: Int J Mol Sci. 2018 Aug 29;19(9):2572. doi: 10.3390/ijms19092572 (PMC6165019; doi:10.3390/ijms19092572)
Supplement: Supplementary file 1 [file ijms-19-02572-s001.pdf]

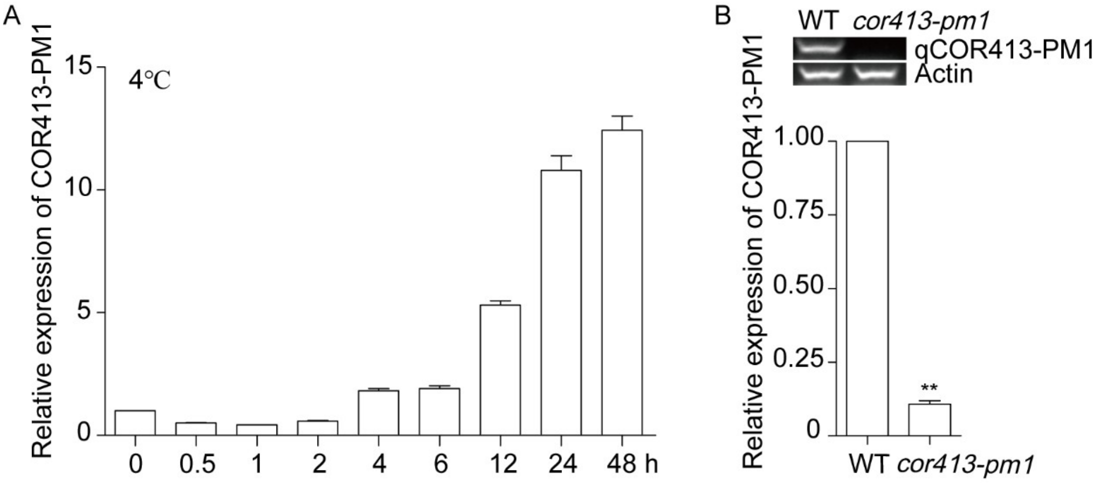

Supplementary Figure S1. Quantitative real-time PCR (qRT-PCR) analysis of *COR413-PM1*. (**A**) Expression pattern of *COR413-PM1* in wild-type (WT) after exposure to 4 °C over different periods of time. Expression level of the gene in the WT at 0 h was set to "1"; (**B**) Expression level of *COR413-PM1* in the WT and *cor413-pm1*. Expression levels of gene in the WT was set to "1". The double asterisks represent significant differences between WT and mutant *cor413-pm1* at $p < 0.01$. Error bars indicate the standard error of the mean (SEM) (n = 4).

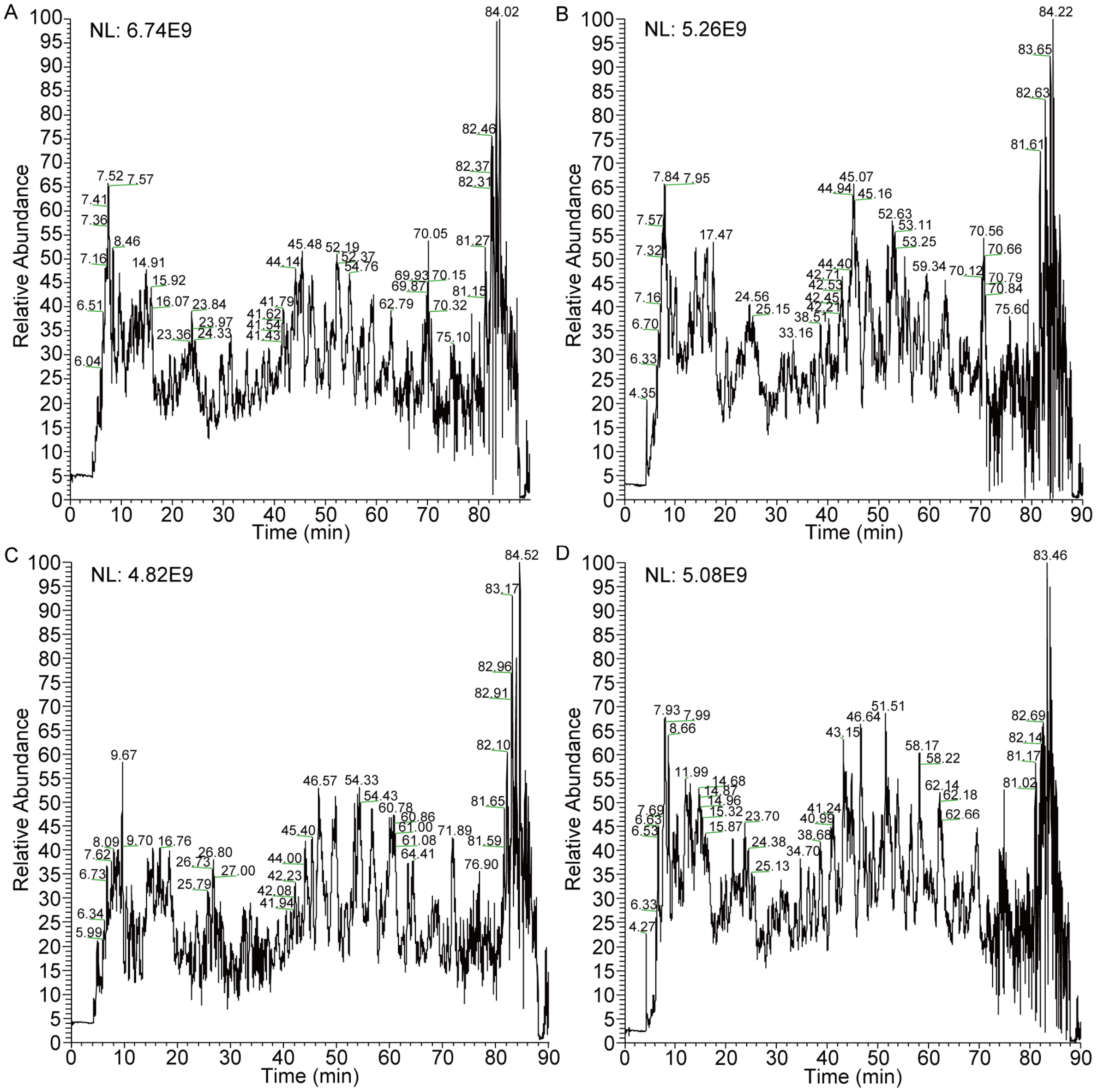

Supplementary Figure S2. The Total Ions Chromatogram (TIC) of (**A**) C-WT, (**B**) C-*cor413-pm1* mutant, (**C**) FT-WT, and (**D**) FT-*cor413-pm1* mutant.

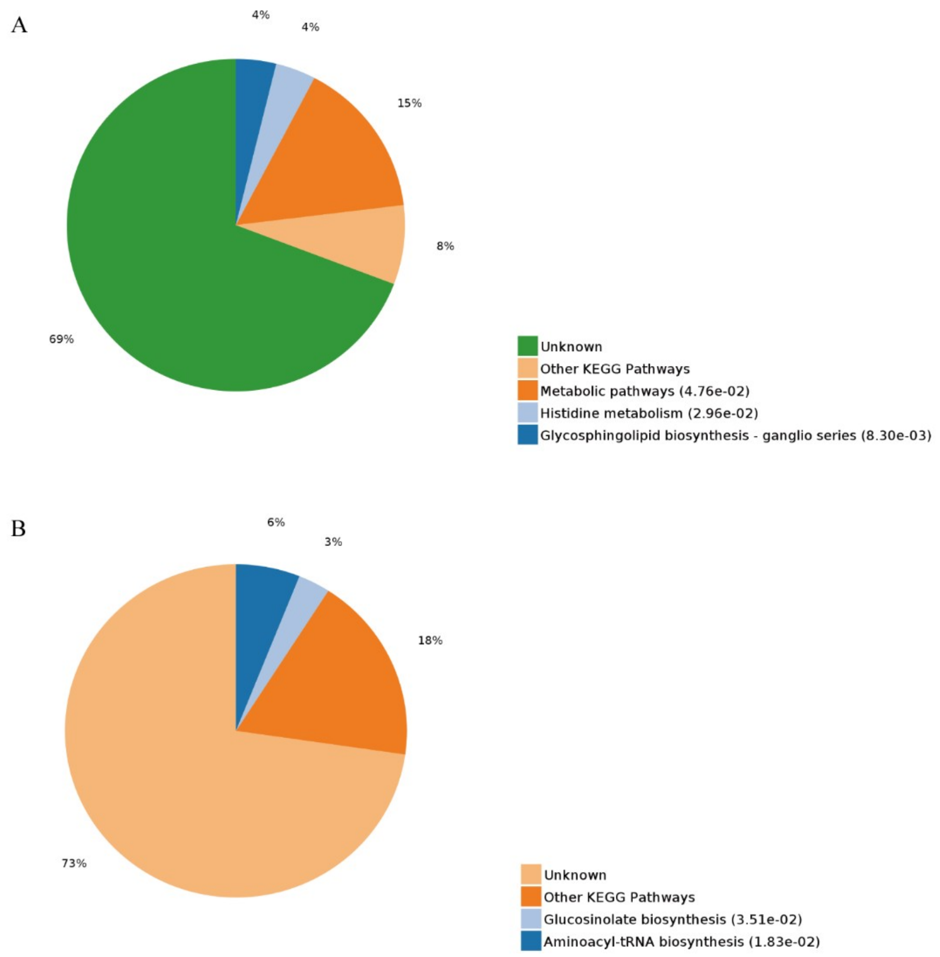

Supplementary Figure S3. Pie charts of KEGG pathways of (**A**) 27 upregulated and (**B**) 34 downregulated differentially abundant proteins (DAPs) associated with *COR413-PM1* gene.

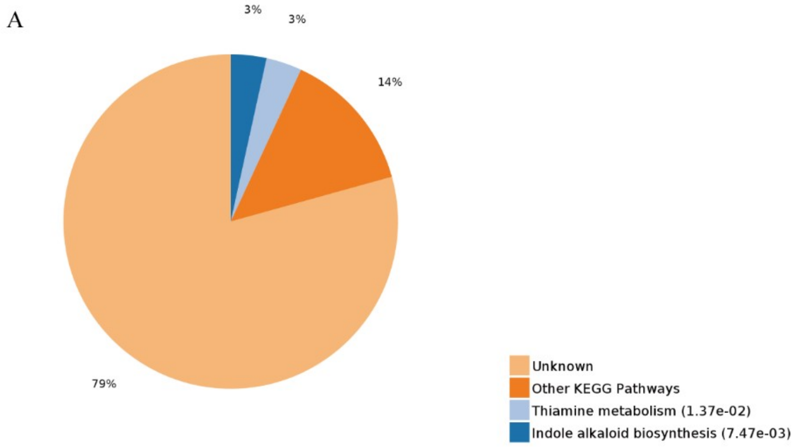

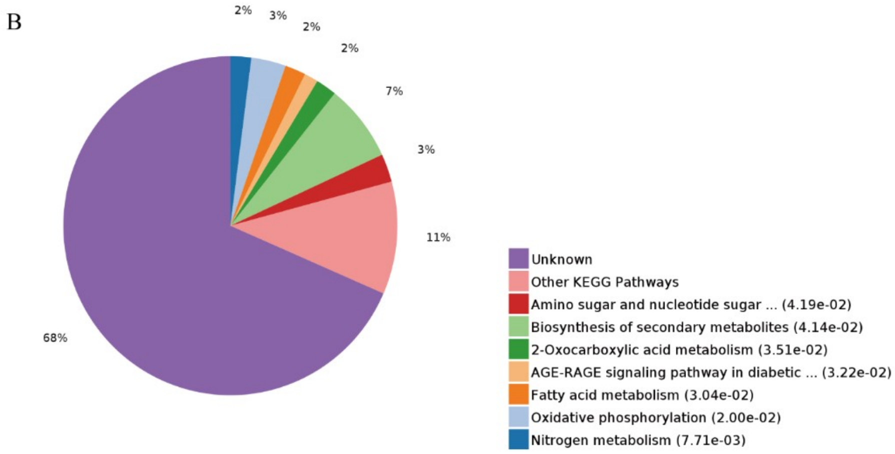

Supplementary Figure S4. Pie charts of KEGG pathways of (A) 30 upregulated and (B) 150 downregulated DAPs associated with freezing treatment.

Supplementary Table S1. Primer sequences used for mutant identification and qRT-PCR analysis.

Primer name	Sense (5′-3′)	Antisense (5′-3′)
COR413-PM1	GTTCGATCCAACACCAACAAG (LP)	CTCAAGACAAATCTTCTGCCG (RP)
LBb1.3	ATTTTGCCGATTTCGGAAC	----
qCOR413-PM1	CCTCGAATGGGTTGCTTCA	CAGCTACGAAAGCAATCCATTT
FAB1	CAATGGGAGGAATGAAGGTC	TGGCACAAGCAGTTGAAATAG
FRK3	GCTCGTCTTGGTGGTTCTTC	ATCAGCACTCGGGTTTCG
CYT1	TCTTGTTGACGAAACCGCTAC	TGCCACCCGATGATACTGC
TPS7	TTTCCTTCACAGTCCCTTCC	TGATTCCCACAGTCCTCCC
LSM4	GCTTCCTCTATCGCTGCTTA	ATCCTCCAAAATCTATCTCCGT
SPSA1	CGCTCTTCTCCATCACTTCTT	GCCAGCCTCTGTGCTTCTT
ASE2	CTCACCGTTTCCTCCGTAAT	CGTCATAATCATCGTTGTCTGC
PYD1	GCTCGCTTTCGGGTATTG	TGCCCTCTGAACTCTTCTATTGT
Actin	GAAATCACAGCACTTGCACC	AAGCCTTTGATCTTGAGAGC

Supplementary Table S2. Important KEGG pathways and proteins in mutant *cor413-pm1* and WT shoots.

Pathway Name/ Protein IDs	Gene Name	Annotation	Q-value	Score
Fatty acid metabolism				
AT1G06290	*ACX3*	acyl-CoA oxidase 3	0.00E+00	8.4029
AT5G15530	*BCCP2*	biotin carboxyl carrier protein 2	8.02E-03	1.2543
AT2G38040	*CAC3*	acetyl co-enzyme a carboxylase carboxyltransferase alpha subunit	0.00E+00	96.63
AT1G74960	*KAS2/FAB1*	a plastidic beta-ketoacyl-ACP synthase II	0.00E+00	6.3661
AT3G11170	*FAD7*	fatty acid desaturase 7	3.81E-04	2.0706
AT1G77590	*LACS9*	long chain acyl-CoA synthetase 9	0.00E+00	6.2493
AT3G55360	*ECR*	enoyl-CoA reductase	8.64E-03	1.2226
AT3G06860	*MFP2*	multifunctional protein 2	0.00E+00	4.3091
AT1G64400	*LACS3*	long-chain acyl-CoA synthetase 3	9.75E-03	1.1225
Starch and sucrose metabolism				
AT5G64570	*BXL4*	beta-D-xylosidase 4	0.00E+00	11.345
AT5G24300	*SS1*	starch synthase 1	0.00E+00	9.3573
AT1G66430	*FRK3*	fructokinases	0.00E+00	11.484
AT5G20280	*SPSA1/SPS1*	sucrose-phosphate synthase	0.00E+00	7.8628
AT1G06410	*TPS7*	trehalose-6-phosphate synthase-like domain	0.00E+00	4.2551
AT4G33220	*PME44*	pectin methylesterase 44	3.79E-03	1.4757
Fructose and mannose metabolism				
AT4G04040	*PFP-BETA2*	beta-subunit phosphofructokinase complex	0.00E+00	4.5972
AT2G22480	*PFK5*	phosphofructokinase 5	3.80E-03	1.4787
AT2G39770	*CYT1/ GMP1*	GDP-mannose pyrophosphorylase	9.26E-03	1.1958
Amino sugar and nucleotide sugar metabolism				
AT3G61130	*GAUT1*	galacturonosyltransferase 1	3.73E-04	1.948
AT1G65590	*HEXO3*	beta-hexosaminidase 3	0.00E+00	2.4767
AT5G17770	*CBR1*	NADH: cytochrome b5 reductase 1	0.00E+00	5.355
Glycolysis / Gluconeogenesis				
AT3G08590	*IPGAM2*	2,3-biphosphoglycerate-independent phosphoglycerate mutase 2	0.00E+00	39.199
AT1G74030	*ENO1*	enolase 1	0.00E+00	7.9623
AT5G52920	*PKP2*	plastidial pyruvate kinase 2	0.00E+00	60.419
AT4G36250	*ALDH3F1*	aldehyde dehydrogenase 3F1	0.00E+00	13.251
AT1G24180	*IAR4*	IAA-conjugate-resistant 4	0.00E+00	38.641
Oxidative phosphorylation				
ATMG00070	*NAD9*	NADH dehydrogenase subunit 9	0.00E+00	24.224
AT3G08610	*AT3G08610*	NADH dehydrogenase ubiquinone 1 alpha subcomplex subunit	3.94E-04	2.2626
AT5G67590	*FRO1*	frostbite 1	0.00E+00	2.9658
AT2G20360	*AT2G20360*	NAD(P)-binding Rossmann-fold superfamily protein	0.00E+00	15.919
AT2G02050	*AT2G02050*	NADH-ubiquinone oxidoreductase B18 subunit	0.00E+00	5.3218
ATCG00430	*NDHK*	subunit K of NADH dehydrogenase	4.12E-03	1.4461
Purine metabolism				
AT4G34740	*ASE2*	glutamine 5-phosphoribosylpyrophosphate amidotransferase	0.00E+00	6.3234
AT2G15430	*NRPB3*	Non-catalytic subunit of nuclear DNA-dependent RNA polymerases II, IV and V	0.00E+00	2.7704
AT2G26230	*UOX*	urate oxidase	0.00E+00	2.8502
RNA degradation				
AT5G27720	*LSM4*	SM-like protein 4	0.00E+00	4.3074
Metabolic pathways				
AT2G30490	*CYP73A5*	cinnamate-4-hydroxylase	0.00E+00	2.741
AT3G06850	*BCE2*	dihydrolipoamide branched chain acyltransferase	3.85E-04	2.1226
AT1G09795	*HISN1B*	ATP phosphoribosyl transferase	0.00E+00	12.864
AT4G35260	*IDH1*	isocitrate dehydrogenase	0.00E+00	28.15

AT2G22450	*RIBA2*	riboflavin biosynthesis protein	0.00E+00	3.1744
AT3G02780	*IPP2*	dimethylallyl diphosphate isomerase activity	0.00E+00	13.603
AT4G32770	*VTE1*	vitamin e deficient 1	4.12E-03	1.437
AT1G08520	*CHLD*	Encodes the CHLD subunit of the Mg-chelatase enzyme	0.00E+00	44.906
AT3G01440	*PNSL3*	photosynthetic NDH subcomplexl 3	0.00E+00	3.8559
AT4G02580	*AT4G02580*	NADH-ubiquinone oxidoreductase 24 kDa subunit	0.00E+00	39.235
AT3G17810	*PYD1*	pyrimidine 1	0.00E+00	51.403
AT4G39640	*GGT1*	gamma-glutamyl transferase	0.00E+00	4.7766
AT1G79440	*ALDH5F1*	mitochondrial succinic semialdehyde dehydrogenase	0.00E+00	6.3666
